# Multi-Dimensional Scaling Analysis of Key Regulatory Genes in Prostate Cancer Using the TCGA Database

**DOI:** 10.3390/genes12091350

**Published:** 2021-08-29

**Authors:** Laura Boldrini, Pinuccia Faviana, Luca Galli, Federico Paolieri, Paola Anna Erba, Massimo Bardi

**Affiliations:** 1 Department of Surgical, Medical, Molecular Pathology and Critical Area, University of Pisa, 56126 Pisa, Italy; pinuccia.faviana@med.unipi.it; 2 Department of Translational Research and New Technologies in Medicine and Surgery, University of Pisa, 56126 Pisa, Italy; lugal71@yahoo.it (L.G.); federico.paolieri@gmail.com (F.P.); paola.erba@unipi.it (P.A.E.); 3 Department of Psychology & Behavioral Neuroscience, Randolph-Macon College, Ashland, VA 23005, USA; massimobardi@rmc.edu

**Keywords:** urological cancer, survival prediction, proliferation, inflammation, stress

## Abstract

Prostate cancer (PC) is a polygenic disease with multiple gene interactions. Therefore, a detailed analysis of its epidemiology and evaluation of risk factors can help to identify more accurate predictors of aggressive disease. We used the transcriptome data from a cohort of 243 patients from the Cancer Genome Atlas (TCGA) database. Key regulatory genes involved in proliferation activity, in the regulation of stress, and in the regulation of inflammation processes of the tumor microenvironment were selected to test a priori multi-dimensional scaling (MDS) models and create a combined score to better predict the patients’ survival and disease-free intervals. Survival was positively correlated with *cortisol* expression and negatively with Mini-Chromosome Maintenance 7 (*MCM7*) and Breast-Related Cancer Antigen2 (*BRCA2*) expression. The disease-free interval was negatively related to the expression of enhancer of zeste homolog 2 (*EZH2)*, *MCM7*, *BRCA2*, and programmed cell death 1 ligand 1 (*PD-L1)*. MDS suggested two separate pathways of activation in PC. Within these two dimensions three separate clusters emerged: (1) *cortisol* and brain-derived neurotrophic factor *BDNF*, (2) *PD-L1* and cytotoxic-T-lymphocyte-associated protein 4 (*CTL4)*; (3) and finally *EZH2*, *MCM7*, *BRCA2*, and *c-Myc*. We entered the three clusters of association shown in the MDS in several Kaplan–Meier analyses. It was found that only Cluster 3 was significantly related to the interval-disease free, indicating that patients with an overall higher activity of regulatory genes of proliferation and DNA repair had a lower probability to have a longer disease-free time. In conclusion, our data study provided initial evidence that selecting patients with a high grade of proliferation and DNA repair activity could lead to an early identification of an aggressive PC with a potentials for metastatic development.

## 1. Introduction

Prostate cancer (PC) is the second most frequent malignancy (after lung cancer) in men worldwide [1]. Global cancer statistics indicated over one million new cases per year causing about 350,000 deaths just in 2018 [2]. Mortality varies dramatically with age and with the stage of detection. When PC is detected later in older patients, mortality increases exponentially, considering that almost 60% of all deaths occur after 65 years of age [3,4]. Therefore, more precise tools to identify early signs of PC or even being able to assess the risk factors in healthy people are essential to reduce the most dramatic consequences of PC. The main problem in detecting PC is that it is often asymptomatic at the early stage; actually, early PC may require minimal or even no treatment [5]. The most frequent complaint is difficulty with urination, increased frequency, and nocturia, all symptoms that may also arise from prostatic hypertrophy.

When further assessments are warranted, the serum prostate-specific antigen (PSA) test is often used to screen for PC [6]. PSA is a glycoprotein normally expressed by prostate tissue. An increasing serum PSA level is usually associated with an advanced stage of the disease and, potentially, poor outcomes [7,8]. However, because men without cancer have also been found with elevated PSA, a tissue biopsy is the standard of care to confirm the presence of cancer. Therefore, Gleason score (GS) is also used in predicting patient outcomes: high GS implies increased tumor aggressiveness and an increased an risk of local and distant tumor spread with a worse prognosis [9,10,11]. However, GS evaluation can be inaccurate for risk stratification considering that sometimes low Gleason tumors may develop into an aggressive disease [12]. Therefore, besides increased PSA serum levels and high GS, other biologic features, such as identifying patterns with extravasated mucin in cases with more complex epithelial bridges, may confer a better prognosis [13].

Considering that PC is a polygenic disease with many gene–gene interactions, several studies have attempted to identify hub genes involved in PC [14]. Some of these works also tried to identify mutations occurring in abnormal prostatic development [15]. It is not unusual to find hundreds of genes highly related to PC progression, often including hubs whose involvement was previously completely unknown [16] Therefore, a detailed analysis of the molecular risk factors can help to understand the connection between genetic mutations and the role of the environment in triggering PC progression [17]. Recently, Seibert and colleagues described the development of a new polygenic hazard score for personalized genetic assessment of individual age associated risk of PC [18] based on single nucleotide polymorphisms. Other researchers used a weighted co-expression network analysis to identify clusters of highly correlated genes as a raking method to find specific monograms able to predict possible outcome for PC [14,19]. Even when studies revealed a large preponderance of specific clusters, such as a molecular taxonomy in which 74% of tumors falling into one of seven subtypes defined by specific gene fusions or mutations [20], patient stratification and outcome remain elusive [21]. Many challenges remain to integrate epidemiological studies with molecular investigations and clinical analyses to gain fundamental insights into how environmental, dietary, and lifestyle influences contribute to the development of PC, and thus a priori models selecting target genes involved in macroscopic functions could shed new light on this complex disease, as previous studies on different types of cancers have revealed [22].

The role of life events, such as dietary conditions and stress, appears to gain traction in recent studies [23]. Intriguingly, previous studies have tested the relationship between stressful life events and PC onset [24]. Cortisol has been showed to have a role in the life events correlated with PSA levels, suggesting an interaction between stress and the hypothalamic-pituitary-adrenal (HPA) axis in relation to cancer [25]. Fabre et al. then reinforced the idea that cortisol may have a role in PC [26]. More recently, the role of BDNF pathway has been investigated in prostate cancer, suggesting the activation of its pathway as a crucial step in disease progression [27].

The role of the tumor microenvironment (TME) as a mediator of metastatic activity has been also extensively evaluated in PC [19,28]. The main hypothesis is that inflammatory injury could prompt carcinogenesis by causing cellular stress and repeated genomic damage [29,30]. However, the role of inflammation in prostate carcinogenesis is still controversial, considering that some evidence showed that prostatic inflammation may confer a protective effect and decrease the rate of subsequent metastasis [31]. Immune checkpoint genes such as *CTLA4* or *PD-L1* have been extensively studied for therapeutic approach, even if with modest efficacy in PC [32]. Nevertheless, including data on the prostate immune microenvironment in a multi-factorial index of cancer aggressiveness could provide essential information in the effort to increase early detection.

One of the extensively studied prognostic markers in PC is cell proliferation activity. It has been shown that the expression of polycomb group protein EZH2 is strongly associated with short progression-free survival and with poor prognosis in many malignancies, including PC [33,34]. Another suggested molecular marker of PC aggressiveness is *MCM7*, a member of a DNA helicase complex involved in the initiation of DNA replication. MCM7, as well as the other five proteins of the family, is overexpressed in several human cancers, such as laryngeal [35], esophageal squamous cell carcinoma [36], and hepatocellular carcinoma [37]. MCM7 and EZH2 are prognostic markers in prostatectomy treated patients [38,39]. Recent studies described the role of EZH2 in DNA repair by regulating the cancer cell fate in response to DNA damage, contributing to DNA double-strand break repair [40,41]. Moreover, a recent work showed that EZH2 expression predicts outcome in patients with *BRCA2*-mutant ovarian tumors by regulating genomic stability at stalled replication forks [42], suggesting a possible connection between EZH2 and BRCA2. Finally, the *Myc* oncogene has a key role in cancer initiation and progression in several cancer types, including PC [43,44]. The regulation of *c-Myc* may be an ideal effective therapeutic target in this disease [45].

In this study, we used a priori approach to select a very specific set of genes involved in the processes detailed above. The advantage of this approach over a more traditional search for cluster of highly related genes [46,47] is related to the ability to look for connections with the behavioral output associated with the disease onset and progression. Therefore, the main aim of this study was to develop an innovative model based on the expression of regulatory genes associated with the stress and immune responses influencing cell proliferation. We used public data from The Cancer Genome Atlas to create a map of association of key regulatory genes involved in proliferation activity, such as *EZH2, MCM7, BRCA2,* and *c-MYC*, in stressful pathway (*BDNF* and *cortisol*), and in inflammation microenvironment (*CTLA4* and *PD-L1*) to test several multi-dimensional scaling models and create a combined gene-cluster to better predict PC. The main weakness of this approach is that we inevitably cannot select all possible combinations of the many genes and cluster of genes that are most certainly involved in this complex disease and its progression.

## 2. Materials and Methods

### 2.1. TCGA Database

This study used data from the public domain and did not require the approval from an ethics committee. The gene expression profiles of patients with prostate cancer were obtained from the TCGA data portal (https://tcga-data.nci.nih.gov/tcga/, accessed on 14 January 2021). Clinical characteristics such as age, latest values of hematic PSA, disease-free interval, survival time, and cancer outcome were also obtained from TCGA data portal. IlluminaHiSeq expression data for 243 patients affects by PC were downloaded and processed.

The Gleason score for each patient was classified in five groups based on their primary and secondary GS (see Table 1). The age at the diagnosis was given in years, whereas time of survival and disease-free interval were in months. Last PSA values were given in ng/mL and ranged from 0 to 323 with a mean of 2.45 ± 22.27 SD.

### 2.2. Statistical Analysis

General Liner Models (GLM) with Tukey’s test as a post hoc test were used for multiple comparisons prior of entering the data into the multivariate models. Pearson’s r was used to calculate the bivariate correlations among clinical output and target gene expression. Survival analyses were performed using the Kaplan–Meier method with the log-rank test for statistical significance. All analyses were considered significant at the α-level = 0.05.

To identify the independent association of all variables (both clinical output and gene expression) a Multi-Dimensional Scaling analysis was run. MDS is a data reduction technique used to reveal the similarities among variables and individual cases in a set of data. Distances between variables were derived looking at partial correlations (i.e., proximities) among variables, which were subsequently used to create a matrix of distance could be displayed graphically. The closer two or more variables are on the map, the more highly correlated they are, while the farther apart they are, the less correlated they are. To arrange the variable into a map sensitive to each individual contribution, a limited lack of fit between the data and the model is inevitable. This lack of fit is known as the s-stress. The values of s-stress range from 0 (perfect fit) to 1 (worst possible fit). Thus, the aim of MDS is to find a map of the variables that minimizes the s-stress. The number of dimensions in a map is linked to the number of latent underlying factors in the dataset, similarly to other procedures like factor analysis. Therefore, the optimal number of dimensions to represent the data is dependent on several factors: the number of variables in the model; the lack of fit (s-stress value), given the number of dimensions; an index of fit of the model (*r*^2^-value); and interpretability of the dimensions. Typically, *r*^2^-values of 0.8 or higher are considered acceptable.

Dimensions revealed by the MDS were entered in a multivariate regression (MR) model to determine if clinical output could be predicted by a linear combination of the target gene expression.

All analyses were performed using SPSS 27.0 (IBM, Armonk, NY, USA).

## 3. Results

### 3.1. TCGA Database

Patients affected by PC (*n* = 243) were extracted from the TCGA database. The average age was 61.36 ± 6.77 SD years, reporting a disease-free interval (DFI) of 34.47 ± 25.90 SD months and overall survival (OS) of 38.12 ± 26.57 SD months. The age of diagnosis was not related to neither disease-free interval (*p* = 0.11) and survival (*p* = 0.11), whereas survival was significantly related to the disease-free interval (*p* = 0.001).

Table 2 showed that neither survival (F_4,238_ = 0.248; *p* = 0.91) nor disease-free interval (F_4,235_ = 1.79; *p* = 0.13) were related to the Gleason score. PSA values were negatively correlated related with the DFI (*r* = −0.18, *p* = 0.007), but it was not related to OS (*p* = 0.09). PSA values was not significantly related to the GS (F_4,211_ = 1.30; *p* = 0.27).

### 3.2. Target Genes

Pearson’s correlations between PSA, disease-free interval, and survival and several target genes are shown in Table 3. Survival was positively correlated with *cortisol* expression (*p* = 0.048) and negatively with *MCM7* (*p* = 0.040) and *BRCA2* (*p* = 0.039). The disease-free interval was negatively related to the expression of *EZH2* (*p* = 0.035), MCM7 (*p* = 0.017) and *BRCA2* (*p* = 0.049), and *PD-L1* (*p* = 0.039). PSA values were positively related to *c-Myc* (*p* < 0.001), *EZH2* (*p* < 0.001), and *MCM7* (*p* = 0.048).

**Figure 1:**
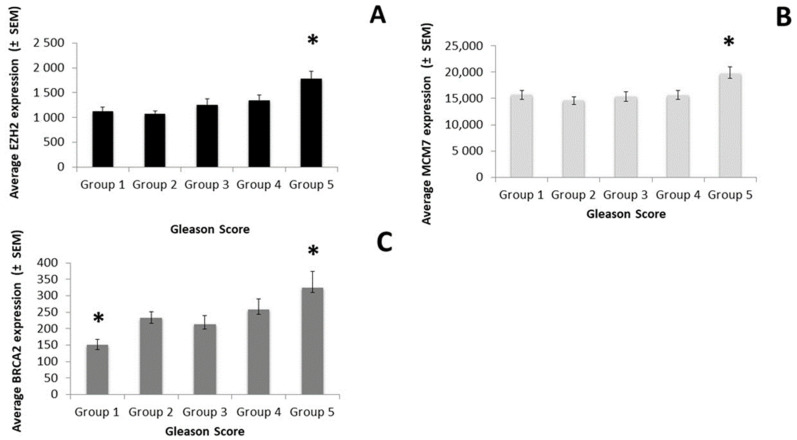
Differences in gene expression by Gleason score classification (Groups 1 though 5). (**A**)—*EZH2* expression, (**B**)—*MCM7* expression, (**C**)—*BRCA2* expression. (*) *p* < 0.05.

The expression of *EZH2* (F_4,238_ = 6.88; *p* < 0.001), *MCM7* (F_4,238_ =4.99; *p* < 0.001), and *BRCA2* (F_4,238_ =3.76; *p* = 0.028) varied significantly by the GS (Figure 1), whereas the other genes did not—although the expression of *BDNF* was close to the significance level (*p* = 0.057).

### 3.3. MDS Analysis

**Figure 2:**
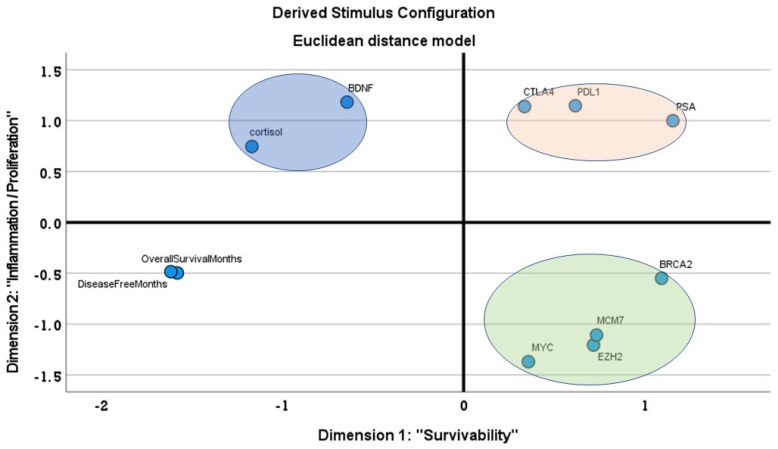
Map of the association among the variables included in the multi-dimensional scaling (MDS) model. Gene expression of target genes and clinical output (Overall Survival and Disease-free Interval) were included in the model. The target genes were grouped in three different clusters according to their main functions. The new dimensions provided by the MDS were named “Survivability” (being the X-axis highly related to the clinical output OS and DFI) and “Inflammation/Proliferation” (two main functions of the clusters of genes identified highly correlated with the Y-axis).

MDS analysis was run to identify the independent association among the target genes and the survival data. S-stress (0.12) and *r^2^* (0.83) were acceptable. The resulting map clearly identified two dimensions, named Survivability and Proliferation/Inflammation axis (Figure 2). The first dimension indicated that higher expression of *cortisol* and BDNF were related to a higher probability to stay free form the disease and, hence, survive; on the other side of the map, higher expressions of *BRCA2* and *MCM7* were related to higher PSA values and, thus, a higher probability of the reappearance of the prostate cancer. The second dimension showed a separation of cluster of proliferation genes (*EZH2*, *c-Myc*, *BRCA2*, and *MCM7*) on one side and inflammation genes (*PD-L1/CTL4*) on the opposite side. This could indicate two separate pathways of activation in PC. Within these two dimensions, three separate clusters emerged: in the top-left quadrant an association between *cortisol* and *BDNF* expression was revealed; in the top-right quadrant the association between *PD-L1* and *CTL4* emerged; finally, in the bottom-right quadrant the target genes *EZH2*, *c-Myc*, *BRCA2*, and *MCM7* clustered together.

### 3.4. Stepwise Multiple Regression

To identify the best predictors of the patient survival, several stepwise multiple regression (SMR) models were run. Firstly, we ran a SMR using only the original values of gene expression. The best model retained the following gene expression: *BRCA2*, *cortisol*, and *BDNF* (Table 4a). Although it was very significant (F_3,239_ = 4.57; *p* = 0.004), the predictive value of this model was quite poor (*r*^2^ = 0.05) thus confirming the complexity of the phenomenon and that no single gene can explain the variability in survival data among patients of PC.

Then, we used the composite indices derived from the MDS. All variables were standardized to take into account the difference in the scale of expression, and each cluster was calculated using the sum of all variables included, so Cluster 1 = *BDN*F + *Cortisol*, Cluster 2 = *PD-L1 + CTL4*, and Cluster 3 = *EZH2 + BRCA2 + cMyc + MCM7*. The best SMR model retained only the last cluster, the composite index derived by Cluster 3 (F_1,241_ = 6.103; *p* = 0.014 in Table 4b). This model had also a much better predictive value than the original one, being able to explain almost 16% of the variance (*r*^2^ = 0.157).

### 3.5. Survival Curve

**Figure 3:**
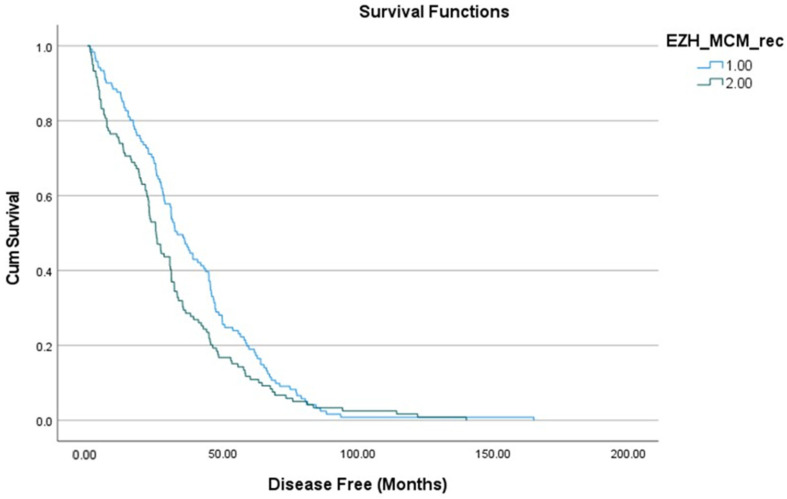
K-M of the cumulative disease-free intervals by Cluster 3.

We entered the three clusters of association shown in the MDS in several Kaplan–Mayer analyses, using the disease-free interval as the time variable. It was found that only Cluster 3 was significantly related to the interval-disease free (Mantel-Cox = 3.69, *p* = 0.05) indicating that patients with an overall higher activity of these 3 key genes (*EZH2*, *BRCA2*, *c-Myc*, and *MCM7*) has a lower probability to have a longer disease-free time (Estimate High: 38.32, 95% CI 33.79 to 42. 85; versus Low: 30.55, 95% CI 25.9 to 35.11 in Figure 3). None of the original gene expression was significantly related to the disease-free interval by its own (all *p*-values > 0.10).

## 4. Discussion

The main aim of this study was to develop a priori model based on the expression of regulatory genes involved in proliferation activity, TME inflammation, and live events related to stress. We found that patients with concurred increased mRNA expression of genes involved in proliferation and DNA repair pathways, such as *EZH2*, *MCM7, BRCA2*, and *c-MYC,* showed to have a more aggressive PC phenotype. We included these genes in our model since several studies based on weighted gene co-expression found that patient outcome and disease progression were related to genes involved in proliferation and DHAN repair [14,19]. While each of these genes has been independently studied in PC progression [37,39], they have never been analyzed together as a combined factor for prognostic use. Our study provided novel evidence that selecting patients with a high grade of proliferation and DNA repair activity could lead to an early identification of an aggressive disease with a potential for metastatic development. Our data specifically linked this cluster of genes with a better prospective for longer disease-free intervals, but not with the overall survival. Although this result seems difficult to explain, since progression-free survival or OS is often used as the endpoint in PC due to its clinical relevance [48,49,50], it is important to remember that in some oncologic disease characterized by a slow growth rate, such as PC, an improvement in DFI appears to be more relevant in the effort of the early identification of aggressive forms of PC. In our study we also merged higher GS, whereas some authors recommend an even higher level of differentiation among the different groups [51], which could influence the overall conclusions.

Several studies have clearly indicated that *c-Myc* is overexpressed at early stages of PC and acts as a key driver of tumorigenesis and disease progression [43]. *c-Myc* overexpression is observed in up to 37% of metastatic PC patients [52] and significantly associated with poor survival [53]. Although the role of *c-Myc* in PC has been intensely studied, little is known concerning the impact of *c-Myc* overexpression in combination with other key regulators in PC progression. Dardenne and colleagues [54] suggested an interaction between *c-Myc* and *EZH2*; they observed high levels of *EZH2* activity in mouse models over-expressing *N-Myc* and in human PC cells, concluding that the histone methyltransferase *EZH2* seems to cooperate with *Myc* in regulating its target genes in neuroendocrine prostate cancer. More recently, Neves Filho and colleagues [55] demonstrated that *EZH2* was up-regulated by *Myc* and associated with high proliferation tumors in diffuse large-B-cell lymphoma. This a priori knowledge was used to include the above genes in our model. The results confirmed that a cluster of genes including *c-Myc*, *EZH2*, *MCM7*, and *BRCA2* could be part of the same regulatory pathway in PC progression. More specifically, *Myc*-mediated stimulation of cell cycle can occur using several parallel mechanisms, also liked to key biological functions such as DNA replication and repair [56].

*MCM7* is one of the heterohexamer MCM helicase complex recruited in initiation of DNA replication. Induced overexpression of *MCM7* is involved in tumor formation and progression in a variety of human malignancies [37,57,58,59,60,61], including PC [38], and its down-regulation results in growth inhibition [62], indicating that *MCM7* is particularly important as a potential biomarker. Here we confirmed that *MCM7* could be a survival predictor for prostate cancer, considering that a shorter disease-free interval was associated with higher *MCM7* and other key proliferation factors expression.

The gene *BRCA2* is involved, together with *BRCA1*, in DNA repair, cell cycle checkpoint regulation and transcription [63]. Several studies have identified DNA repair gene signatures as effective predictors of PC progression and outcome [47,64] It is currently understood that the normal protein product of *BRCA2* gene is important in double-strand DNA repair by maintaining genomic integrity, and that once this gene is mutated or altered, DNA damage may not be repaired properly, likely leading to the occurrence of cancer. How DNA repair is affected by homeostatic processes involving life habits and stress responses is not sufficiently understood. It is known that cells undergo a constant burden of damage that can be increased dramatically by environmental agents and social events, generating many of the same DNA lesions that are a hallmark of cancer progression [65]. Our results appear to indicate that life-base and cellular-base DNA damages contribute to PC cancer following two separate pathways. Accumulated evidence has demonstrated that the expression level of *BRCA2* is altered in breast and ovarian cancer, offering a potentially important tool for use in cancer management [66]. *BRCA2* mutations are well recognized in PC [67,68], associated with a poor prognosis [69,70,71], and with a high response rate to PARP inhibitors in patients not responding to standard treatments [72]. However, no data exist regarding *BRCA2* expression level, and its crosstalk within a network of proteins in PC.

Intriguingly, *BRCA2* and other DNA repair genes seem to be influenced by circadian modulation [73], and diet habits [74], probably in connection with diurnal hormonal modulation. Since PC is also a hormone-dependent tumor, nutrients potentially affect tumor pathogenesis and progression [75,76] through various mechanisms including inflammation, cortisol-mediated stress effects, and the action of sex hormones [77]. Recently, Li and colleagues [27] investigated the role of BDNF in prostate cancer, demonstrating that the BDNF pathway is crucial for disease progression. Our integrated data confirmed that a higher expression of *BDNF* and *cortisol* were related to a greater probability of survival. Chronic stress is known to promote tumor progression in several cancer models [78], and a growing body of evidence suggests that the stress response machinery is an important mediator during tumorigenesis and metastasis [79,80,81,82,83,84]. Steroid hormones are critical factors in mediating the stress-cancer relationship; stress-related hormones have been shown to be involved in accelerating cell proliferation and tumor growth in PC [85]. However, our understanding of the mechanisms through which stress contributes to cancer development and progression is incomplete. Our data of elevated *cortisol* mRNA levels confirmed a previous study reporting increased circulating levels of cortisol in PC patients [26], suggesting a possible relationship between stress and cancer. 

Cancer is a multistep process that requires cells to acquire specific characteristics in order to evolve into a malignant phenotype; it is unlikely that stress alone can provide cells with such traits, however, stress can influence a wide variety of cellular functions, facilitating deregulation of pro- and anti-proliferative cellular processes. Without doubts, the regulation of mRNA expression by stress signals (both internal and external) represents an emerging and promising field of study in PC and it needs further analysis. Another route of action, which has been extensively studied in recent years, is focused on immune function and its well-established relationship with stress [86,87]. Reprogramming innate immune cells is one of the main ways by which the tumor controls the surrounding microenvironment in order to promote its growth [88]. A complex series of interactions between immune cell types and non-tumor cells within the TME heavily impact tumor progression, invasion, and metastasis. A chronic inflammatory prostate microenvironment seems to drive prostate carcinogenesis and progression [89]. A growing number of trials are ongoing with the immune checkpoint antibodies in prostate cancer. PC grows slowly compared to other types of malignancies, which allows it to be an ideal candidate for immunotherapy. However, various clinical trials by immunotherapy (anti-PD1 and anti-CTLA4) have only shown modest clinical outcomes in comparison to other cancers. Particularly, TME in prostate lesions may be unsuitable for tumor infiltrating immune cells with anti-tumor activities, leading to limited efficacy of immunotherapy [90]. Bishop et al. [91] showed variations in *PD-L1* expression in prostate tumors, suggesting that the levels of immune checkpoint molecule expression vary in different stages of PC progression and our data of *PD-L1* and *CTLA4* mRNA expression confirmed this mechanism. These variations in immune checkpoint molecules expression in prostate tumors need more studies in order to assess their putative clinical use as potential indicator of checkpoint immunotherapy and tumors progression.

Until recently, Gleason score and serum PSA levels represented the most important predictive factors during PC management [92]. The recommended grade grouping based on GS is routinely adopted. However, issues remain regarding quantification of high-grade patterns and the mostly indolent behavior of these tumors. Moreover, the PSA level is not able to discriminate aggressive or advanced prostate cancer, particularly at PSA levels below 20 ng/mL [93,94], and it has a high false-positive rate. Considering that the currently available methods cannot provide accurate parameters for the prediction of aggressive potential in prostate cancer, tissue-based biomarkers could be used as prognostic markers [95], and hence the need of a more sophisticated level of analysis as exemplified in our study.

Although the results in the current study are promising and confirm the necessity of integrated genetic markers to enhance early detection of aggressive forms of PC, it would be important to validate our conclusions in experimental settings that go beyond the scope of using the TCGA database. For starter, multiple pathways of a priori selected genes should be tested to find the best combination as a predictive tool for PC. Although the gene selected in this work are clearly implicated in the development of PC, many other key regulatory genes could be involved in the functional pathway of PC, alternative models would be certainly improved by including different alternative pathways.

## 5. Conclusions

The novelty of our approach warrants further investigation on the role of proliferation and DNA repair genes in the role of PC, and demonstrates that the role of the TME and life events, such as stress, can be key factors in detecting PC progression with a high clinical impact.

## Figures and Tables

**Table 1:** Number of patients by Gleason score and group classification.

Classification	Primary GS	Secondary GS	N. of Cases
Group 1	3	3	25
Group 2	3	4	73
Group 3	4	3	47
Group 4	4	4	29
Group 5	4, 5	4, 5	69

GS: Gleason score.

**Table 2:** Average ± SD of clinical output data (prostate-specific antigen (PSA) scores, Age at diagnosis in years, overall survival (OS) in months, and disease-free interval (DFI) in months) by Gleason score classification (Group 1 through 5).

	Gleason Score													
	Group 1		Group 2		Group 3		Group 4		Group 5		Total		F	*p*
	Mean	SD	Mean	SD	Mean	SD	Mean	SD	Mean	SD	Mean	SD		
OS	34.91	27.63	38.46	29.14	37.61	23.86	41.55	24.46	36.89	26.36	37.85	26.53	0.25	0.91
DFI	34.91	27.63	37.85	29.52	35.24	22.67	39.85	24.48	27.68	22.94	34.47	25.91	1.79	0.13
PSA	0.08	0.20	0.09	0.40	0.51	2.37	0.81	2.33	8.01	41.93	2.45	22.27	1.30	0.27
Age	59.24	7.92	60.53	7.06	61.68	6.07	61.10	6.07	63.03	6.52	61.40	6.76	2.01	0.1

**Table 3:** Correlation among clinical output (prostate-specific antigen (PSA) scores, Age at diagnosis, overall survival (OS), and disease-free interval (DFI) and target gene expression. Significant values are in bold. (*) *p* < 0.05; (**) *p* < 0.01.

		Age	OS	DFI	*MYC*	*EZH2*	*MCM7*	*BRCA2*	*PDL1*	*cortisol*	*BDNF*	*CTLA4*
PSA	*r*	−0.011	−0.115	−0.184 **	0.322 **	0.634 **	0.135 *	−0.032	0.008	−0.045	−0.044	−0.004
	*p*-value	0.868	0.092	0.007	0.000	0.000	0.048	0.636	0.905	0.514	0.521	0.953
Age	*r*		−0.104	−0.105	0.016	0.057	0.107	0.067	0.168 **	−0.011	0.129 *	0.096
	*p*-value		0.105	0.105	0.799	0.379	0.097	0.295	0.009	0.866	0.044	0.137
OS	*r*			0.911 **	−0.018	−0.096	−0.132 *	−0.133*	−0.125	0.127 *	−0.077	−0.009
	*p*-value			0.000	0.783	0.134	0.040	0.039	0.051	0.048	0.231	0.885
DFI	*r*				0.008	−0.136 *	−0.155 *	−0.127 *	−0.133 *	0.092	−0.078	−0.036
	*p*-value				0.897	0.035	0.017	0.049	0.039	0.154	0.229	0.576

**Table 4:** (a) Stepwise regression of the disease-free interval (DFI) by gene expression. (b) Stepwise regression of the disease-free interval (DFI) by gene-clusters.

**(a** **)**						
**Model**		**Unstandardized Coefficients**		**Standardized Coefficients**	***t***	***p***
		**B**	**Std. Error**	**Beta**		
1	(Constant)	41.266	2.357		17.505	0.000
	*BRCA2*	−1.366 × 10^−5^	0.000	−0.133	−2.077	0.039
2	(Constant)	37.395	3.001		12.463	0.000
	*BRCA2*	−1.407 × 10^−5^	0.000	−0.137	−2.152	0.032
	*cortisol*	5.340 × 10^−6^	0.000	0.131	2.063	0.040
3	(Constant)	39.324	3.100		12.686	0.000
	*BRCA2*	−1.331 × 10^−5^	0.000	−0.129	−2.050	0.042
	*cortisol*	8.093 × 10^−6^	0.000	0.198	2.840	0.005
	*BDNF*	−1.116 × 10^−5^	0.000	−0.156	−2.226	0.027
**(b)**						
**Model**		**Unstandardized Coefficients**		**Standardized Coefficients**	***t***	***p***
		**B**	**Std. Error**	**Beta**		
1	(Constant)	34.401	1.650		20.852	0.000
	CLUS3	−2.077	0.749	−0.177	−2.774	0.006
Dependent Variable: Disease Free (Months)						

## Data Availability

Not applicable.
